# Indazole Derivatives Against Murine Cutaneous Leishmaniasis

**DOI:** 10.3390/ph18081107

**Published:** 2025-07-25

**Authors:** Niurka Mollineda-Diogo, Yunierkis Pérez-Castillo, Sergio Sifontes-Rodríguez, Osmani Marrero-Chang, Alfredo Meneses-Marcel, Alma Reyna Escalona-Montaño, María Magdalena Aguirre-García, Teresa Espinosa-Buitrago, Yeny Morales-Moreno, Vicente Arán-Redó

**Affiliations:** 1 Centro de Bioactivos Químicos, Universidad Central “Marta Abreu” de Las Villas, Santa Clara 54800, Villa Clara, Cuba; niurkam@uclv.cu (N.M.-D.); omarrero@uclv.edu.cu (O.M.-C.); ameneses@uclv.edu.cu (A.M.-M.); yenymm@uclv.edu.cu (Y.M.-M.); 2 Grupo de Bio-Quimioinformática and Facultad de Ingeniería y Ciencias Aplicadas, Universidad de Las Américas, Quito 170125, Ecuador; 3 Instituto de Investigaciones Biomédicas, Universidad Nacional Autónoma de México—Consejo Nacional de Humanidades, Ciencias y Tecnologías (CONAHCYT), Ciudad de México 14080, Mexico; oigresergio@gmail.com; 4 Unidad de Investigación UNAM-INC, Facultad de Medicina, Universidad Nacional Autónoma de México—Instituto Nacional de Cardiología Ignacio Chávez, Ciudad de México 14080, Mexico; almaescalona@comunidad.unam.mx (A.R.E.-M.); maguirre@unam.mx (M.M.A.-G.); 5 Departamento de Microbiología y Parasitología, Facultad de Farmacia, Universidad Complutense de Madrid, Pza. Ramón y Cajal s/n, 28040 Madrid, Spain; emb.teresa@gmail.com; 6 Facultad de Farmacia, Universidad San Pablo-CEU, CEU Universities, Urbanización Montepríncipe, Boadilla del Monte, 28660 Madrid, Spain; 7 Instituto de Química Médica del Consejo Superior de Investigaciones Científicas de España, Juan de la Cierva 3, 28006 Madrid, Spain; uvejotaran@gmail.com

**Keywords:** neglected tropical diseases, 3-alkoxy-1-benzyl-5-nitroindazole, *Leishmania amazonensis*, cutaneous leishmaniasis, BALB/c mice, in vivo antileishmanial activity

## Abstract

**Background/Objectives**: Leishmaniasis is a zoonotic and anthropozoonotic disease with significant public health impact worldwide and is classified as a neglected tropical disease. The search for new affordable treatments, particularly oral and/or topical ones that are easy to administer and have fewer side effects, remains a priority for the scientific community in this field of research. In previous investigations, 3-alkoxy-1-benzyl-5-nitroindazole derivatives showed remarkable in vitro results against *Leishmania* species, and predictions of absorption, distribution, metabolism, excretion, and toxicity properties, as well as pharmacological scores, of the compounds classified as active were superior to those of amphotericin B, indicating their potential as candidates for in vivo studies. Therefore, the aim of the present study was to evaluate the in vivo antileishmanial activity of the indazole derivatives NV6 and NV16. **Methods**: The compounds were administered intralesionally at concentrations of 10 and 5 mg/kg in a BALB/c mouse model of cutaneous leishmaniasis caused by *Leishmania amazonensis*. To evaluate the efficacy of the compounds, indicators such as lesion size, ulcer area, lesion weight, and parasitic load were determined. Amphotericin B was used as a positive control. **Results**: The compound NV6 showed leishmanicidal activity comparable to that observed with amphotericin B, with a significant reduction in lesion development and parasite load, while NV16 caused a reduction in ulcer area. **Conclusions**: These results provide strong evidence for the antileishmanial activity of NV6 and support future studies to improve its pharmacokinetic profile, as well as the investigation of combination therapies with other chemotherapeutic agents currently in use.

## 1. Introduction

Leishmaniasis is a zoonotic and anthropozoonotic disease [1,2], classified by the World Health Organization (WHO) among the ten most impactful neglected tropical diseases affecting global public health [3]. It mainly occurs in three clinical forms: cutaneous (localized and disseminated), mucocutaneous, and visceral (kala azar) [4,5].

Cutaneous leishmaniasis (CL) is considered non-fatal [4], is the most common form, accounts for 95% of cases worldwide, and is widespread in tropical and subtropical regions [6,7]. Healing can take between 5 months and 20 years in affected individuals [4], and it is characterized by the appearance of skin ulcers, which can cause significant physical disability and social stigma [8]. Despite its prevalence, this clinical form receives limited attention and ranks among the lowest priorities on global health agendas, making it the most neglected of the neglected diseases for which there is no investment in the development of new therapies [6,9]. Mucocutaneous leishmaniasis (MCL) is restricted to Latin America, affecting the mucous membranes of the nose, mouth, and throat, while visceral leishmaniasis (VL) is the most serious and can be fatal if left untreated, causing lesions in internal organs such as the liver and spleen, with an estimated annual mortality rate of 20,000 to 40,000 cases [10].

The control of leishmaniasis remains challenging due to the high diversity of *Leishmania* species and strains, as well as the limited understanding of the immunological mechanisms governing *Leishmania*-specific memory responses and protective immunity. This lack of knowledge has hindered the development of an effective vaccine against the disease [11]. In addition, the susceptibility to drugs of the species prevalent in different endemic areas affects the clinical efficacy of available treatments [12]. The drugs currently used in antileishmanial therapy, such as pentavalent antimony derivatives, amphotericin B, miltefosine, and pentamidine, have high toxicity and cause severe side effects [8], which often lead to early discontinuation of treatment, therapeutic failure, and decreased efficacy due to the development of drug resistance [13]. The search for new therapeutic options, particularly oral or topical formulations that are easy to administer and better tolerated, remains a critical priority for researchers in this field [14,15].

A novel class of indazole derivatives has attracted the attention of researchers worldwide due to its biological and pharmacological properties [16,17,18,19,20,21,22]. The chemistry of indazole derivatives is of growing interest, and over the last decade, interesting aspects of this heterocyclic system have been discovered [20,23,24], such as their in vitro activity against *Trypanosoma cruzi* [17,19,22], *Trichomonas vaginalis* [16,17], and several *Leishmania* species [18,20,21,25].

The 1-substituted-2-benzyl-5-nitroindazolin-3-one and 3-alkoxy-1-benzyl-5-nitroindazole derivatives have shown promising in vitro leishmanicidal activity [20,21]. However, its in vivo efficacy remains largely unexplored, primarily due to the optimization of the compounds identified as active, with the aim of enhancing their pharmacokinetic properties [19], and the current regulations on the use of laboratory animals. Therefore, it is essential to advance preclinical studies using various biological models to evaluate their antileishmanial potential. These studies will confirm the antileishmanial activity of the most promising compounds identified in in vitro tests, thus facilitating the selection of potential candidates for the development of new drugs against leishmaniasis.

Derivatives of 3-alkoxy-1-benzyl-5-nitroindazoles have shown potent and selective inhibitory activity against intracellular amastigotes of *Leishmania amazonensis* (IC_50_ = 0.43–5.6 µM and SI = 11–129), *L. infantum* (IC_50_ = 1.2–3.8 µM and SI = 16–20), and *L. mexicana* (IC_50_ = 1–2.2 µM and SI = 14–48) [21]. In addition, predictions of Absorption, Distribution, Metabolism, Excretion, Toxicity (ADMET) [26] and drug score [27] for the compounds classified as active indicate improved physicochemical properties, intestinal absorption, and oral bioavailability compared to amphotericin B, further supporting their potential as candidates for in vivo studies [21].

On the other hand, BALB/c mice are a highly susceptible inbred strain to *L. amazonensis* [28,29,30] in which a predominant Th2 immune response impairs the ability to eliminate the intracellular parasite [31,32], allowing rapid spread of the disease. This model is ideal for determining the in vivo efficacy of candidate drugs against *L. amazonensis* [33,34,35,36], whereby an active compound would be one capable of causing a significant reduction in lesion size and parasite load, even in the absence of complete cure [37,38,39].

**Figure 1:**
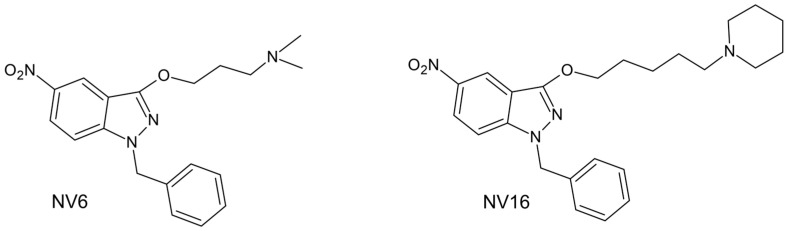
Structure of 3-alkoxy-1-benzyl-5-nitroindazole derivatives NV6 and NV16.

Two derivatives of 3-alkoxy-1-benzyl-5-nitroindazoles, NV6 (3-((1-benzyl-5-nitro-1*H*-indazol-3-yl)oxy)-*N*,*N*-dimethylpropan-1-amine) (IC_50_ = 0.43 µM and SI = 71) and NV16 (1-benzyl-5-nitro-3-((5-(piperidin-1-yl)pentyl)-1*H*-indazole)) (IC_50_ = 0.17 µM and SI = 129) (Figure 1), were selected for an in vivo efficacy study due to their promising activities against intracellular amastigotes of *L. amazonensis* [21]. The compounds were evaluated in a murine model of experimental cutaneous leishmaniasis at the base of the tail of BALB/c mice, caused by *L. amazonensis*, with the aim of determining their potential as candidates for antileishmanial drugs.

## 2. Results

The 30%, 20%, and 10% DMSO solutions administered subcutaneously every other day for 14 days did not cause any observable signs of local skin irritation, discomfort, or weight loss in the treated mice. Due to the low solubility of the nitroindazolines under study, a 30% DMSO solution was chosen as the vehicle.

In relation to the Maximum Tolerated Dose (MTD), in general, local irritation was observed in the clinical examination at a dose of 20 mg/kg for both compounds (NV6 and NV16). Although not severe, these effects prompted a dose reduction to 10 mg/kg. No deaths were observed during and after treatment.

**Figure 2:**
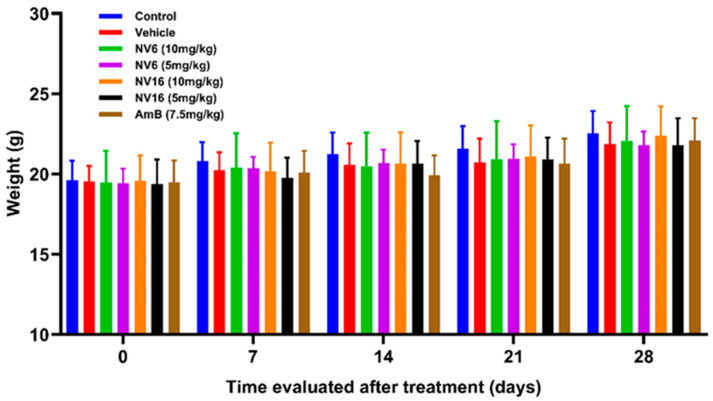
Evolution of body weight in the treatment groups from the start to the end of the experiment.

All administered doses were well tolerated, as indicated by the absence of irritation, local discomfort, or significant changes in body weight (*p* > 0.05, Figure 2) throughout this study. Moreover, all treated animals survived.

**Figure 3:**
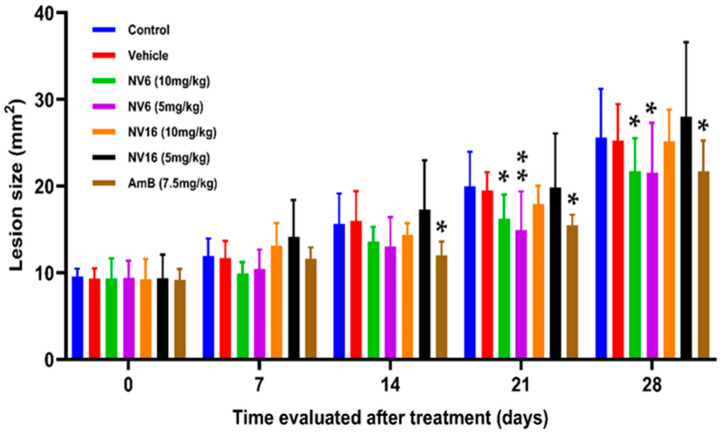
Effect of treatment with NV6 and NV16 at doses of 10 mg/kg and 5 mg/kg of live weight on lesion size (*: *p* < 0.05, **: *p* < 0.01).

Throughout the 28-day study period (Figure 3 and Figure S1), no therapeutic effect on lesion size was observed in the vehicle control group as no statistically significant differences (*p* > 0.05) were observed relative to the control group. Similarly, NV16 (10 and 5 mg/kg) did not demonstrate any therapeutic effect on lesion size. In contrast, NV6 (10 and 5 mg/kg) and amphotericin B produced comparable reductions in lesion size.

Intralesional administration of NV6 at doses of 10 mg/kg and 5 mg/kg during the first week of treatment showed no significant differences (*p* > 0.05) compared to the control, vehicle, and amphotericin B groups. After 14 days, the lesion size in the amphotericin B-treated group was significantly reduced compared to both the control (*p* = 0.042) and vehicle control (*p* = 0.025) groups. Although lesion size was reduced after 14 days of treatment with NV6, this effect was not statistically significant (*p* > 0.05) relative to the control and vehicle groups.

By day 21, lesion size in the group receiving NV6 at 10 mg/kg was significantly reduced relative to the control group (*p* = 0.037). Likewise, a dose of 5 mg/kg led to a statistically significant reduction (*p* < 0.01) in lesion size compared to the control group (*p* = 0.0051), and significantly different lesion size from the vehicle control group (*p* = 0.010). After the same time, the treatment with amphotericin B produced a significant reduction in lesion size compared to the control (*p* = 0.012) and the vehicle control (*p* = 0.024) groups.

At the end of the experiment (28 days), the reduction in lesion size was statistically different for NV6 at 10 mg/kg relative to the control (*p* = 0.028) and vehicle (*p* = 0.045) groups. The same compound at 5 mg/kg reduced lesion size relative to the control (*p* = 0.023) and vehicle (*p* = 0.038) groups, while the reduction in lesion size caused by amphotericin B significantly differed from control (*p* = 0.028) and vehicle (*p* = 0.047) groups.

**Figure 4:**
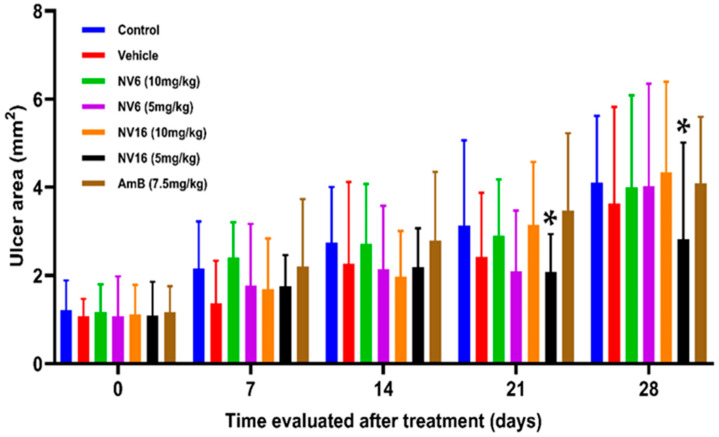
Effect of treatment with NV6 and NV16 at doses of 10 mg/kg and 5 mg/kg of live weight on the ulcer area (*: *p* < 0.05, compared to the amphotericin B group).

On the other hand, there were no statistically significant differences in ulcer area between groups (*p* > 0.05) during the first two weeks of treatment (Figure 4 and Figure S1). Subsequently, significant differences were observed between the NV16 (5 mg/kg) and amphotericin B groups (*p* = 0.049) on days 21 and 28 post-treatment initiation.

**Figure 5:**
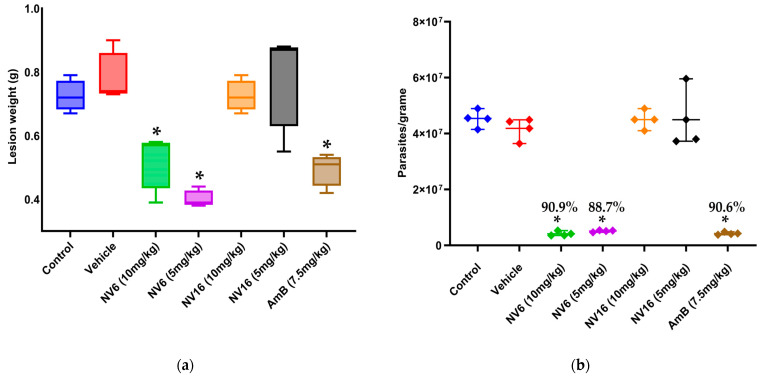
Effect of treatment on lesion weight (**a**) and parasite load (**b**) one week after completion of treatment. (*: *p* < 0.05, compared to the control group). Bars represent geometric means, and error lines indicate minimum and maximum values. Data labels indicate the percent reduction in parasite load relative to untreated control mice.

The weight of the lesions at the end of the experiment (Figure 5a) was significantly lower (*p* < 0.05) for treatment with NV6 at both doses and with amphotericin B (7.5 mg/kg) compared to control and vehicle control groups. Parasite load in lesions was also significantly reduced (*p* < 0.05) in mice treated with NV6 (10 mg/kg), NV6 (5 mg/kg), and amphotericin B (7.5 mg/kg) compared to the control group (Figure 5b and Figure S2). Lesion weight and parasite load were statistically comparable (*p* > 0.05) among mice treated with NV6 (10 mg/kg), NV6 (5 mg/kg), and amphotericin B.

## 3. Discussion

The in vivo study was successfully conducted, the treatments were well tolerated, and no changes in body weight were observed, indicating a lack of toxicity. The weight ranges throughout this study were consistent with those established for the strain, age, and sex of the rodents used [40].

A previous report stated that compound NV6 (3-((1-benzyl-5-nitro-1*H*-indazol-3-yl)oxy)-*N*,*N*-dimethylpropan-1-amine) was active against promastigotes and amastigotes of three *Leishmania* species. Its activity against *L. amazonensis* amastigotes met the selection criteria [41] for classification as promising (IC_50_ = 0.43 µM and SI = 71) [21] and against the same stage in *L. infantum* (IC_50_ = 3.8 µM and SI = 16) and *L. mexicana* (IC_50_ = 2.2 µM and SI = 14), showed growth-inhibitory activity comparable to that of several synthetic compounds currently under investigation as new antileishmanial alternatives, such as aminoquinoline derivatives (IC_50_ = 8.1–15.6 μM) [42] and ravuconazole (IC_50_ = 5.11 μM) [43]. In the present study, NV6 at concentrations of 10 and 5 mg/kg demonstrated leishmanicidal activity in vivo, with significant reductions in lesion size and weight, and parasite load, comparable to that of amphotericin B.

From a clinical point of view, amphotericin B is the most effective drug available for treating any form of leishmaniasis, and is used in cases that are refractory to other antileishmanial drugs [44,45]. Amphotericin B and pentavalent antimony compounds are the two antileishmanial drugs frequently used as positive controls in rodent experiments to evaluate new compounds or treatments for leishmaniasis. However, due to the progressive development of strains resistant to antimonial drugs and the need to test recent clinical isolates, the use of amphotericin B is preferable, since parasitological resistance has been rarely reported [46]. The efficacy of amphotericin B as a positive control depends on the dose used and the timing of treatment initiation [46]. Previous studies report its low efficacy as a positive control in experimental animals infected with *Leishmania* when doses lower than 4 mg/kg are used, which is associated with a high rate of parasite multiplication in the lesion [47], or when treatment is started late and the lesions have been developing for several weeks [46]. In a previous study with amphotericin B at a dose of 7.5 mg/kg [46], a reduction in lesion size and infection rates similar to those observed in our study was reported.

The compound NV16 (1-benzyl-5-nitro-3-((5-(piperidin-1-yl)pentyl)oxy)-1*H*-indazole) had no therapeutic effect on lesion size and parasitic load, with a progression pattern similar to that observed in the control and vehicle groups, despite the fact that it showed superior in vitro activity and selectivity index (IC_50_ = 0.17 µM, SI = 129) against intracellular amastigotes of *L. amazonensis* than that of NV6 [21].

Studies on intracellular amastigotes are an initial step in the drug development process that helps prioritize chemical compounds for subsequent in vivo studies [48], but the results of in vivo experiments, as in this case, do not always correlate with in vitro activities. Poor macrophage penetration may limit the efficacy of many compounds. These derivatives share 85% structural similarity, and the structure-activity relationships have been previously discussed [21]. NV16 has a pentyl group attached to a bulky saturated heterocyclic amine (piperidine) as a substituent at the 3-O position of the indazole scaffold, while the substituent in NV6 is a tertiary amine attached by a propyl group (Figure 1). NV6 has a lower molecular weight and fewer rings in its structure than NV16; consequently, it may have better chances of rotation and internalization in the macrophage membrane than NV16 [21,49]. The therapeutic failure of NV16 could be related to the limited availability of the compound inside the macrophage; moreover, a previous study reported that this compound has a less favorable octanol-water partition coefficient (log *p* = 5.03) compared to NV6 (log *p* = 3.32) [21].

The vehicle control (DMSO 30%) and NV16 at 5 mg/kg showed similar ulcer area behavior at the beginning of treatment, an aspect that could be related to the immunomodulatory properties described for DMSO [50]. However, only NV16 at 5 mg/kg showed a significant reduction (*p* < 0.05) in the area of the ulcer compared to amphotericin B. The compound had no effect on the area of the ulcer at the highest concentration tested. In this sense, everything indicates that the solubility of NV16 is key to its action in reducing the ulcer area, which influences its absorption and distribution within extracellular tissues. Skin ulcers caused by *Leishmania* and exposed to the environment are frequently infected secondarily with other microorganisms, such as fungi and bacteria, which complicates the healing of the lesions [4]. In this sense, the antibacterial properties of indazole derivatives have been described previously [51], and this result suggests that the observed effect may be attributable to the known antibacterial properties of this class of compounds [51,52]. This effect may seem contradictory when compared to its therapeutic failure to reduce lesion size, but it could be the case that NV16 faces fewer barriers to exert its antibacterial activity than its antileishmanial effect in the ulcer.

Considering our results, compound NV6 can be selected to advance to future stages of development, while NV16 is not considered suitable for further development. Although no clinical healing of the lesions was observed, the statistical significance of the therapeutic effect is nonetheless relevant, since the animal model used is very sensitive to the parasite and typically shows progressive lesion development [33]. In this regard, once chemotherapy is discontinued, the remaining parasites in the lesion multiply and cause the lesions to grow [53]. Furthermore, definitive clinical cure has rarely been reported in BALB/c mice infected with *L. amazonensis* after treatment with a chemotherapeutic agent, whether clinically approved or in the experimental phase. In general, as in our research, a delay in lesion growth and a decrease in parasite load have been demonstrated [38,39,54]. A notable exception is the combination of amiodarone and miltefosine, which resulted in the clinical cure in mice, sustained throughout one year of follow-up [55].

Additional previous evidence supports the selection of NV6 as a candidate for future development. In recent years, various topical treatments with new drug candidates or drug combinations against cutaneous leishmaniasis have been evaluated in BALB/c mice infected with various *Leishmania* species [54]. Studies with intralesional administration of compounds such as essential oils from *Chenopodium ambrosioides* [56] inhibited the progression of infection and reduced the parasite load in experimentally infected mice compared to controls treated with Glucantime as a positive control, producing results comparable to those obtained in our study. Intralesional administration of antileishmanial compounds reduces the risks of toxicity and modulates host immune responses involved in controlling parasite dissemination, while it appears to improve the compounds’ in vivo efficacy. For most infectious diseases, an ideal drug would be able to eliminate the causative agent in the animal model. However, in line with clinical results and the immune response to *Leishmania* in BALB/c mice [54], it is reasonable to expect a reduction in lesion size and parasite load in this animal model for a promising drug candidate, even in the absence of complete clinical resolution.

In summary, the results of the indazole derivative NV6 in the experimental model used for cutaneous leishmaniasis provide robust evidence supporting this compound as an antileishmanial agent. In future research, other vehicles and formulations should be evaluated to optimize pharmacokinetics, biodistribution, cellular uptake, and the amount of compound transported to infected cells. Combinations of NV6 with antileishmanial drugs currently in clinical use can also be explored.

## 4. Materials and Methods

### 4.1. Chemical Compounds

The nitroindazolines NV6 (3-((1-benzyl-5-nitro-1*H*-indazol-3-yl)oxy)-*N*,*N*-dimethylpropan-1-amine) and NV16 (1-benzyl-5-nitro-3-((5-(piperidin-1-yl)pentyl)oxy)-1*H*-indazole) were synthesized from 1-benzyl-5-nitroindazol-3-ol via alkylation reactions. The details of their synthesis have been previously published [16], as well as their in vitro activity against *L. amazonensis*, cytotoxicity in mouse peritoneal macrophages, and ADMET properties [21]. Amphotericin B sodium deoxycholate (Julio Trigo López Pharmaceutical Laboratory, Havana, Cuba) was used as a positive control. Prior to use, amphotericin B was dissolved in sterile water, and doses were adjusted according to the body weight of the mice.

### 4.2. Parasites

The reference strain MHOM/BR/77/LTB0016 of *L. amazonensis*, donated by the Pedro Kouri Institute of Tropical Medicine, Cuba, was used for the experiments. The parasites were isolated from skin lesions of previously infected BALB/c mice and maintained at 26 °C in RPMI–1640 medium (Gibco, Paisley, Scotland, UK) supplemented with antibiotics (penicillin sodium 200 IU/mL and streptomycin 200 μg/mL, SIGMA, St. Louis, MO, USA) and 10% heat-inactivated fetal bovine serum (FBS, SIGMA, St. Louis, MO, USA) (56 °C, 30 min). Parasites were kept in the exponential growth phase by passaging every 3–4 days. Stationary phase cultures (5 days) were used to infect the animals.

### 4.3. Animals

Female BALB/c mice, 8–10 weeks old and 18–20 g body weight, supplied by the National Centre for the Production of Laboratory Animals (CENPALAB, Mayabeque, Cuba) and certified for genetic and sanitary quality, were used in the experiments. They were kept under a 12 h/12 h light/dark regime, with room temperature of 20–25 °C and a relative humidity of 60–65%. Mice were housed in T3 polycarbonate cages at a rate of 8 mice per cage and were handled by qualified personnel. They were humanely killed by CO_2_ inhalation at the end of the studies. All procedures complied with Directive 2010/63/EU of the European Parliament on the protection of animals used for scientific purposes (22 September 2010) to ensure the least possible suffering. The experimental protocol was previously approved by the Institutional Ethics Committee and the Scientific Council of Center for Bioactive Chemicals, Marta Abreu Central University of Las Villas (SC-CBQ-UCLV), Santa Clara, Cuba (Approval No: IEC-SC-CBQ-01/2024, Date of Approval: 1 September 2024).

### 4.4. Vehicle Selection

Subcutaneous administration using dimethyl sulfoxide (DMSO) as a vehicle is the most commonly used method for administering drugs to rodents [57]. Adverse reactions are primarily due to its concentration and route of administration [58]. Considering the above and with the aim of determining the concentration of DMSO that can be used as a vehicle without causing signs of inflammation, necrosis, or skin irritation, DMSO test solutions were prepared in PBS (phosphate-buffered solution) (pH = 7) from pure DMSO (Sigma-Aldrich, St. Louis, MO, EUA). Five treatment groups of two mice each were formed, and concentrations of 50%, 40%, 30%, 20%, and 10% were tested in a final volume of 50 µL. Two administration sites were used on the back: one received daily injections, while the other received injections every other day. Mice were observed at 2 h and 24 h after subcutaneous injection to identify signs of local irritation such as skin erythema, inflammatory reactions, distension, or necrosis at the application sites [57].

### 4.5. Determination of the Maximum Tolerated Dose

The nitroindazolines selected for this study were characterized by their low solubility. At doses above 20 mg/kg, in 50 µL of 30% DMSO, microprecipitates of the compound were observed after preparation of the injectable solution. For the maximum tolerated dose (MTD) study, groups of five mice were formed and started at 20 mg/kg body weight, administered subcutaneously on alternate days for 14 days. The animals were observed daily to assess the appearance of signs of toxicity and the occurrence of deaths. They were also weighed at 4-day intervals to assess weight behavior as a general indicator of toxicity. In cases where deaths occurred, treatment was halted and restarted in a new group at half the previous dose using the same experimental protocol.

### 4.6. In Vivo Assay

Female BALB/c mice (n = 8) were infected subcutaneously at the base of the tail with 10^7^ stationary-phase promastigotes of *L. amazonensis* per mouse. Once lesions of approximately 10 mm^2^ had developed (4 weeks after inoculation), the mice were randomly assigned to seven experimental groups. This study was conducted in a double-blind manner. Intralesional treatment with NV6 or NV16 (10 mg/kg and 5 mg/kg) was administered on alternate days in a volume of 50 μL [59]. The vehicle used to dissolve the compounds was a solution of DMSO (30%). One group was treated with amphotericin B (positive control), at a dose of 7.5 mg/kg in 50 µL, intraperitoneally, every other day [46]. Two negative control groups were used, one receiving no treatment (control group) and another one treated with the vehicle (vehicle control group). All mice received a total of seven doses for 14 days.

Body weight, lesion size, and ulcer area were measured weekly for 4 weeks. Lesion size and ulcer area were determined using a lesion segmentation and area measurement software [60], which allows measurements by processing photographic images. The general condition of the mice (pain, skin irritation, stress or loss of well-being) was monitored during the treatment.

At the end of the experiment, four mice per treatment group were sacrificed, the lesions were removed and weighed, and the parasite load was determined using the limiting dilution assay (LDA) [61]. The percentages of infection reduction were calculated using the following formula:

% PIR = (PLC − PLT) × 100/PLC

where

% PIR: Percentage infection reduction
PLC: Parasite load of the control group
PLT: Parasite load of the treated group

Impressions of the injuries of four mice per group were taken, and after Giemsa staining, images were captured using an Accu-Scope model 3015 optical microscope, with a 100× objective (using immersion oil) and equipped with a 3.2-megapixel UCMOS03100KPA digital camera (with an optical magnification of 50× due to the combination of the objective and the camera lens), to visualize the differences between the treatment groups.

### 4.7. Statistical Analysis

Repeated measures analysis of variance (ANOVA) and Fisher’s Least Significant Difference (LSD) test were used to determine the evolution of lesion size over the study period. Lesion weights were compared using ANOVA and Dunnett’s test (versus untreated control and vehicle control). Parasite loads were compared using the Kruskal–Wallis test and the post hoc distribution-free multiple comparison test. Values of *p* < 0.05 were considered statistically significant. Analyses were performed with GraphPad Prism software (version 10.2.0, https://www.graphpad.com/, accessed on 5 November 2024).

## 5. Conclusions

The compound NV6 (3-((1-benzyl-5-nitro-1*H*-indazol-3-yl)oxy)-*N*,*N*-dimethylpropan-1-amine) exhibited therapeutic efficacy against *L. amazonensis* comparable to that of amphotericin B, with significant reductions in lesion growth and parasite load at doses of 10 and 5 mg/kg body weight. These findings provide strong evidence of the antileishmanial potential of 3-alkoxy-1-benzyl-5-nitroindazole derivatives, particularly NV6, and support future studies aimed at optimizing their pharmacokinetic properties. Next steps should include evaluating NV6 activity in other biological models, exploring different forms and routes of administration, as well as in combination with other currently available antileishmanial agents, to maximize their therapeutic potential.

## Data Availability

The raw data supporting the conclusions of this article will be made available by the authors on request. The data are not publicly available due to privacy reasons.

## Supplementary Materials

The following supporting information can be downloaded at https://www.mdpi.com/article/10.3390/ph18081107/s1, Figure S1: Development of skin lesions due to leishmaniasis in the treatment groups. Images are representative of each experimental group; Figure S2: Lesion prints stained with Giemsa, representative of the different treatment groups. The images show macrophages infected with intracellular amastigotes of *L. amazonensis* (optical magnification 50×); the extracellular amastigotes are a consequence of the excision of the lesion. A (control group), B (vehicle control), C (NV6 10 mg/kg), D (NV6 5 mg/kg), E (NV16 10 mg/kg), F (NV16 5 mg/kg), and G (AmB 7.5 mg/kg).
