# Supplementary material for: Indazole Derivatives Against Murine Cutaneous Leishmaniasis
Source: Pharmaceuticals (Basel). 2025 Jul 25;18(8):1107. doi: 10.3390/ph18081107 (PMC12389175; doi:10.3390/ph18081107)
Supplement: Supplementary file 1 [file pharmaceuticals-18-01107-s001.zip › pharmaceuticals-3767973-supplementary.pdf]

Indazole Derivatives Against Murine Cutaneous Leishmaniasis

Niurka Mollineda-Diogo[1], Yunierkis Pérez-Castillo[2*], Sergio Sifontes-Rodríguez[3], Osmani Marrero-Chang[1], Alfredo Meneses-Marcel[1], Alma Reyna Escalona-Montaño[4], María Magdalena Aguirre-García[4], Teresa Espinosa-Buitrago[5,6], Yeny Morales-Moreno[1], Vicente Arán-Redó[7]

[1] Centro de Bioactivos Químicos, Universidad Central "Marta Abreu" de Las Villas, Santa Clara, Villa Clara, 54800 Cuba. NMD: niurkam@uclv.cu; OMC: omarrero@uclv.edu.cu AMM: ameneses@uclv.edu.cu; YMM: yenymm@uclv.edu.cu

[2] Grupo de Bio-Quimioinformática and Facultad de Ingeniería y Ciencias Aplicadas, Universidad de Las Américas, Quito 170125, Ecuador. YPC: yunierkis@gmail.com

[3] Instituto de Investigaciones Biomédicas, Universidad Nacional Autónoma de México—Consejo Nacional de Humanidades, Ciencias y Tecnologías (CONAHCYT), Ciudad de México 14080, Mexico. SSR: oigresergio@gmail.com

[4] Unidad de Investigación UNAM-INC, Facultad de Medicina, Universidad Nacional Autónoma de México—Instituto Nacional de Cardiología Ignacio Chávez, Ciudad de México 14080, Mexico. AREM: almaescalona@comunidad.unam.mx; MMAG maguirre@unam.mx

[5] Departamento de Microbiología y Parasitología, Facultad de Farmacia, Universidad Complutense de Madrid, Pza. Ramón y Cajal s/n, 28040 Madrid, Spain. TEB: emb.teresa@gmail.com

[6] Facultad de Farmacia, Universidad San Pablo-CEU, CEU Universities, Urbanización Montepríncipe, Boadilla del Monte, 28660 Madrid, Spain. TEB: emb.teresa@gmail.com

[7] Instituto de Química Médica del Consejo Superior de Investigaciones Científicas de España, Juan de la Cierva 3, 28006 Madrid, Spain. VAR: uvejotaran@gmail.com

* Correspondence: YPC: yunierkis@gmail.com

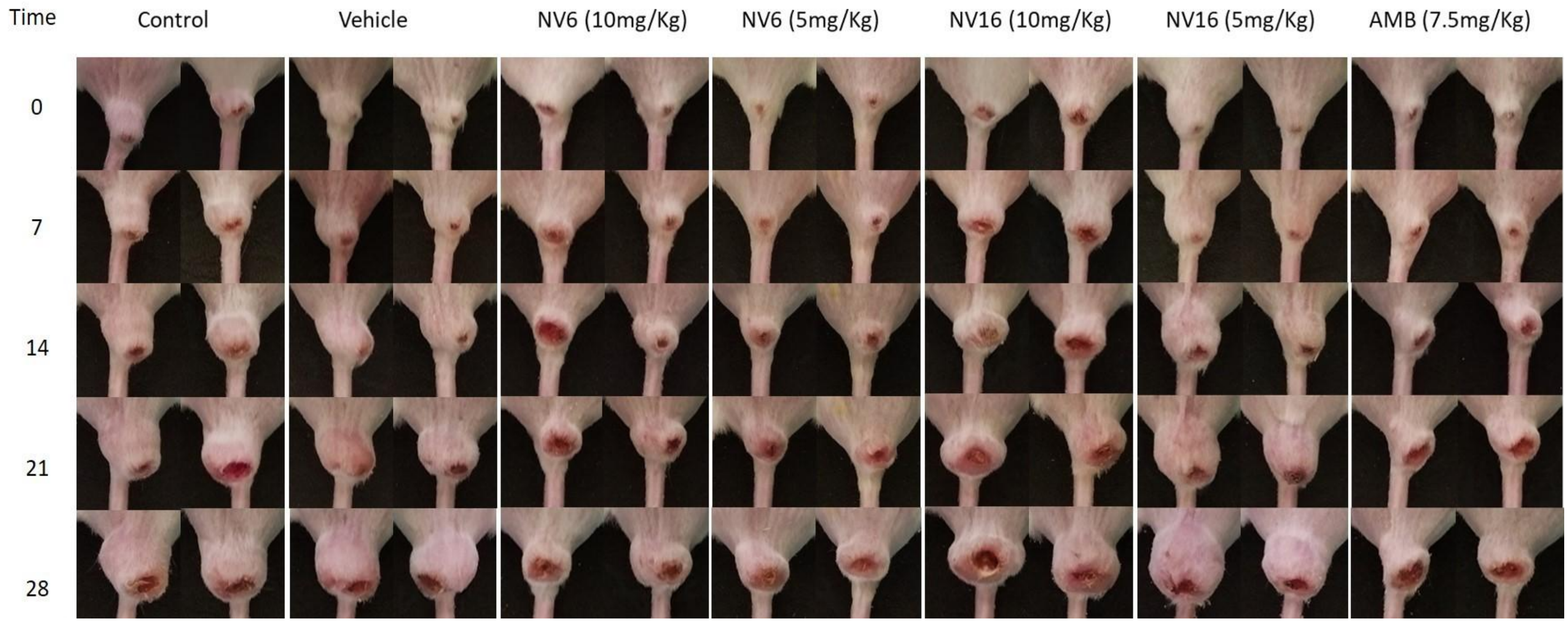

Figure S1: Development of skin lesions due to leishmaniasis in the treatment groups. Images are representative of each experimental group

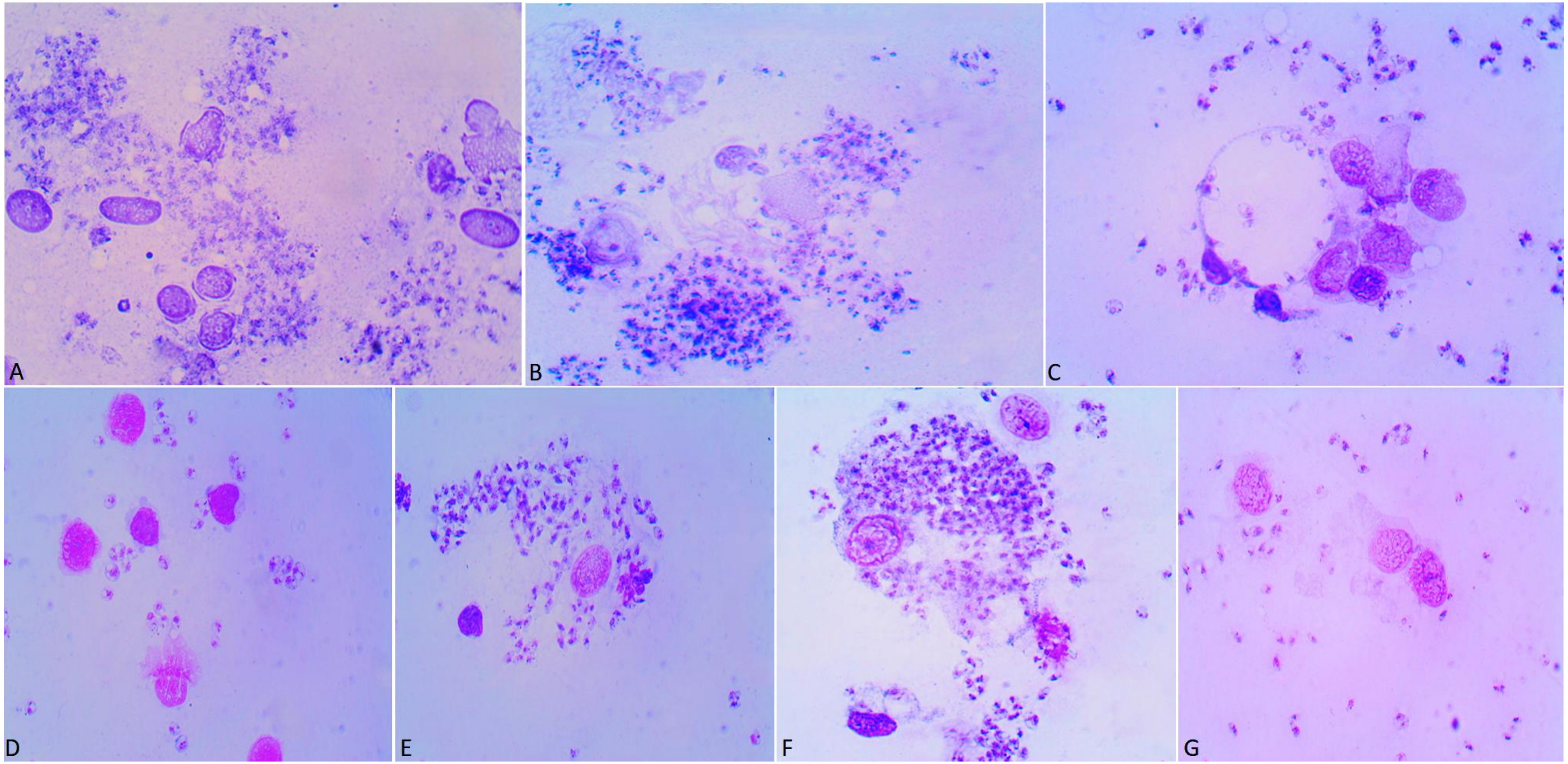

Figure S2: Lesion prints stained with Giemsa representative of the different treatment groups. The images show macrophages infected with intracellular amastigotes of *L. amazonensis*; the extracellular amastigotes are a consequence of the excision of the lesion. A (control group), B (vehicle control), C (NV6 10mg/kg), D (NV6 5mg/kg), E (NV16 10mg/kg), F (NV16 5mg/kg), and G (AmB 7.5mg/kg).
